# Harnessing the translational power of bleomycin model: new insights to guide drug discovery for idiopathic pulmonary fibrosis

**DOI:** 10.3389/fphar.2023.1303646

**Published:** 2023-11-30

**Authors:** Annalisa Murgo, Fabio Bignami, Giuseppina Federico, Gino Villetti, Maurizio Civelli, Angelo Sala, Daniela Miglietta

**Affiliations:** ^1^ Global Research and Early Development, Chiesi Farmaceutici S.p.A., Parma, Italy; ^2^ Dipartimento di Scienze Farmaceutiche, Università degli Studi di Milano, Milan, Italy

**Keywords:** forced vital capacity, diffusion factor for carbon monoxide, Nintedanib, Pirfenidone, Ashcroft score, automated histology imaging analysis, metalloproteinase-7, Broncho alveolar lavage fluid

## Abstract

**Background:** Idiopathic pulmonary fibrosis (IPF) is a chronic, progressive, age-related interstitial lung disease (ILD) with limited therapeutic options. Despite the wide variety of different *in vivo* models for IPF, these preclinical models have shown limitations that may significantly impair their translational potential. Among the most relevant limitations are the methodologies used to assess the efficacy of anti-fibrotic treatments, that are not the ones used in humans. In this scenario, the goal of the work presented in this paper is to provide translational relevance to the bleomycin (BLM)-induced pulmonary fibrosis mouse model, introducing and validating novel readouts to evaluate the efficacy of treatments for IPF.

**Methods:** The BLM model was optimized by introducing the use of functional assessments such as the Forced Vital Capacity (FVC) and the Diffusion Factor for Carbon Monoxide (DFCO), that are respectively the primary and secondary endpoints in clinical trials for IPF**,** comparing them to more common readouts such as lung histology, improved by the application of Artificial Intelligence (AI) to detect and quantify fibrotic tissue deposition, and metalloproitenase-7 (MMP-7), a clinical prognostic biomarker.

**Results:** Lung function measurement and DFCO changes well correlated with Ashcroft score, the current gold-standard for the assessment of pulmonary fibrosis in mice. The relevance and robustness of these novel readouts in the BLM model was confirmed by the results obtained testing Nintedanib and Pirfenidone, the only drugs approved for the treatment of IPF patients: in fact, both drugs administered therapeutically, significantly affected the changes in these parameters induced by BLM treatment, with results that closely reflected the efficacy observed in the clinic. Changes in biomarkers such as MMP-7 were also evaluated, and well correlated with the modifications of FVC and DFCO.

**Conclusion:** Novel functional readouts such as FVC and DFCO can be efficiently used to assess pathology progression in the BLM-induced pulmonary fibrosis mouse model as well as compound efficacy, substantially improving its translational and predictivity potential.

## Introduction

IPF is a fatal interstitial lung disease of unknown origin with an average life expectancy of 3–5 years after diagnosis if untreated (Lederer and Martinez, 2018; Raghu et al., 2018). The reported incidence of IPF has been estimated to be 10 cases per 100,000 population per year, in Europe and North America (Sauleda et al., 2018)**,** but with the population aging worldwide, the impact of IPF on patients and healthcare providers is expected to steadily increase in the future.

To date, Pirfenidone and Nintedanib are the only two therapies approved for IPF worldwide (Spagnolo et al., 2015; Spagnolo et al., 2018; Raghu et al., 2022) but both drugs have limited efficacy in preventing disease progression and despite improving the quality of life, they are also associated with tolerability issues (Bando et al., 2016; Galli et al., 2017). The incomplete understanding of the disease and the limitations of current treatments make IPF a disease with a high unmet medical need, strongly requiring novel treatment approaches.

Despite the extensive variety of experimental models of lung fibrosis (Tashiro et al., 2017), one of the most challenging aspects of drug discovery for IPF is the identification of new molecules using preclinical animal models that can translate effectively to the clinic (Jenkins et al., 2017; Carrington et al., 2018). As a result, the translational potential of newly developed therapies is limited and most drugs that were successful in preclinical models then failed in clinical trials (Spagnolo and Maher, 2017).

One possible explanation could be the limited use of clinically relevant endpoints (i.e., lung function evaluations), in preclinical IPF models, given that the most used readouts in such models are histological analysis and collagen content (Carrington et al., 2018). Pulmonary function tests (PFT’s) routinely implemented in clinics are the first step in the diagnosis of IPF and in the monitoring of its progression (Raghu et al., 2022) and the forced vital Capacity (FVC) is indeed considered the most predictive for restrictive lung disease (Nathan et al., 2020)**.** An absolute or relative decline in % predicted FVC ≥10% is associated with mortality (Durheim et al., 2015; Paterniti et al., 2017)**,** and FVC decline is selected as primary endpoints in the pivotal phase 3 trials of antifibrotic therapies (Noble et al., 2011; King et al., 2014; Richeldi et al., 2014). Despite its importance in the clinic, measuring pulmonary function in mice remains challenging and therefore not often used (Irvin and Bates, 2003). However, the evaluation of FVC in the mouse model of pulmonary fibrosis accompanied by histological readouts may prove capable of improving confidence in new anti-fibrotic candidates.

Additional read-outs, such as the diffusing capacity of the lungs for carbon monoxide (DLCO), also known as the diffusion factor for carbon monoxide (DFCO), are commonly used as secondary endpoint in clinical trials assessing the efficacy of novel treatments for IPF. DFCO is a variable that directly reflects structural changes in the lung and is almost always reduced in IPF patients at the time of initial evaluation (Cortes-Telles et al., 2014). To support the importance of DFCO measurements for the clinical appraisal of IPF, it must be noted that DFCO values strongly correlate with both dyspnea (Swigris et al., 2012) and survival (Hamada et al., 2007)**.** Despite its ability to provide very reproducible measurements, sensitive to a host of pathologic changes in the lung phenotype, DFCO has been only seldom evaluated to assess lung pathologies in mice, in part because the procedure is complex and/or requires dedicated equipment.

The aim of the present study was therefore to introduce novel functional readouts such as FVC and DFCO in an established BLM mouse model (Stellari et al., 2017; Ruscitti et al., 2020), and validate these novel endpoints by testing the effects of the two currently approved drugs, Pirfenidone and Nintedanib. To further improve the model, an automated image analysis using an artificial intelligence (AI) approach (Pontis et al., 2019), was also introduced and validated. Finally, to demonstrate the robustness of these novel functional readouts, we correlated these data with a biomarker involved in the extra cellular matrix (ECM) remodeling, such as metalloproteinase-7 (MMP-7), one of the most promising prognostic biomarkers in IPF, for which it is reported a correlation with disease severity, as assessed by FVC and DFCO, in several clinical studies (Inoue et al., 2020).

Overall, the final aim of the present work was to provide supporting evidence that evaluating functional parameters may indeed enhance the translational potential of the mouse BLM model, building a bridge between the preclinical experience and the clinical practice and reducing the attrition rate of much needed novel treatments for IPF.

## Methods

### Animals

Male C57BL/6J mice (∼25 g, 6–7 weeks old), were supplied by Envigo RMS Italy, San Pietro al Natisone (Udine, Italy). Prior to use, animals were housed 5 per cage and acclimatized for at least 5 days to the local vivarium conditions (room temperature: 20°C-24°C relative humidity: 40%–70%; 12-h light-dark cycle), having free access to standard certified rodent chow and controlled tap water. Animals were checked for abnormalities or signs of health problems after arrival in the animal facility and were acclimatized for a period of at least 1 week before starting any experimental procedure.

During the experimental phase, mice were given daily high calories dietary supplement (DietGel Recovery purchased by Clear H20, Westbrook, ME, United States) and sterile sunflower seeds (Sunflower kernels w/o hulls, purchased by ssniff-Spezialdiäten GmbH, Soest, DE) in addition to the standard rodent chow, to limit body weight loss and percentage of mortality. Moreover, mice health conditions (pain rating by visual analogue scale) were checked every day, while the body weight was measured every 2 days throughout the duration of all the studies. All the experimental procedures involving experimental animals were conducted in an AAALAC (Association for Assessment and Accreditation for Laboratory Animal Care) certified facility and were approved by the local ethics committees and authorized by the Italian Ministry of Health in full compliance with the international European ethics standards of directive 2010/63/EU, Italian D.L. 43 26/2014, and the revised “Guide for the Care and Use of Laboratory Animals” (Committee for the Update of the Guide for the Care and Use of Laboratory Animals and National Research Council, 2010).

### Experimental design

**FIGURE 1:**
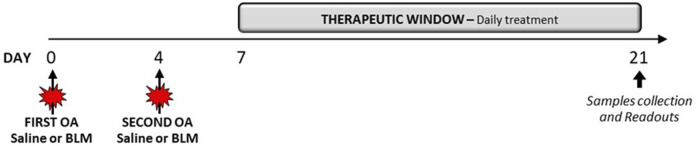
Experimental protocol of a therapeutic BLM OA Study.

After the acclimatization period, mice were instilled via oropharyngeal aspiration (OA) under 4% isoflurane anesthesia with saline or BLM (BAXTER Oncology GmbH, Germany) solution (0.03 IU/mouse), at day 0 and day 4. Nintedanib Esylate (50 mg/kg), Pirfenidone (200 mg/kg), or Vehicle (1% Tween 80 in milliQ water) were administered orally twice daily for 2 weeks following a therapeutic protocol starting at day 7 after the first administration of BLM, until day 21 (Figure 1).

Twenty-one days after the first administration of BLM, mice were deeply anesthetized intraperitoneally with a mixture of ketamine (100 mg/kg) and xylazine (10 mg/kg). After that, animals were tracheostomized and intubated with a cannula to assess DFCO measurements. At the end of the procedure, animals were injected intraperitoneally with Pancuronium (1 mg/kg) to allow the lung functionality analysis and to collect all the readouts as described below.

### Measurement of diffusion factor for carbon monoxide (DFCO)

According to the published procedure (Limjunyawong et al., 2015), the gas chromatograph (microGC fusion gas analyzer-Inficon), using a molecular sieve column with helium as carrier gas, was set up to measure peaks for nitrogen, oxygen, neon, and carbon monoxide. At the start of each experimental day, prior to making measurements of the samples from mice, a 2 mL sample was taken directly from a gas mixture bag containing approximately 0.3% Ne, 0.3% CO, and balance air, and used to calibrate the gas chromatograph. A single module Micro GC Fusion configured with a 10 m Molsieve 5A column was used to analyze gas standard. The column temperature was operated isothermally at 130°C for 40 s 21 days after the first BLM OA administration, mice were anesthetized with ketamine (100 mg/kg) and xylazine (10 mg/kg), tracheostomized with a stub needle cannula (18 G) and DFCO was measured as previously described (Limjunyawong et al., 2015). Briefly after withdrawing 0.8 mL of gas from a gas mixture bag containing approximately 0.3% Ne, 0.3% CO, and balance air, the syringe was connected to the tracheal cannula and the lung was quickly inflated. After 9 s 0.8 mL of exhaled air were withdrawn and injected into the gas chromatograph for analysis. A second sampling was immediately carried out to calculate an average of the two DFCO measurements. DFCO was calculated as 1–(CO_9_/CO_c_)/(Ne_9_/Ne_c_), where c and 9 subscripts refer to concentrations of the calibration gases injected and the gases removed after a 9 s breath hold time, respectively.

### Lung function tests (FVC measurement)

21 days after the first BLM administration and following the DFCO assessment, respiratory system mechanics and Pressure-Volume relationships were measured using the FlexiVent System (SCIREQ Inc., Montreal Qc, Canada) (Vanoirbeek et al., 2010). Each mouse, using an 18-gauge metal endotracheal cannula, was connected to the FlexiVent and ventilated at a respiratory rate of 150 breaths/min and tidal volume of 10 mL/kg against a positive end-expiratory pressure of 3 cmH_2_O to achieve a mean lung volume close to that during spontaneous breathing. To prevent spontaneous breathing mice also received Pancuronium bromide 1 mg/kg intraperitoneally. FlexiVent software version 8.1 was used to perform the perturbations. The negative pressure-driven forced expiration (NPFE) maneuver was carried out by inflating the mouse lungs to a pressure of 30 cm H_2_O over 1 s, holding this pressure for 2 s before connecting the animal’s airways to the negative pressure reservoir (−50 cm H_2_O) for 2 s. The NFPE maneuver should mimic spirometry in humans generating outcomes similar to those obtained in IPF patients, such as FVC. All maneuvers and perturbations were performed until three acceptable measurements (coefficient of determination ≥0.95) were achieved. An average of the three measurements was calculated and depicted per mouse.

### Biological samples collection and processing

After completion of functional assessments, BALF and lung samples were collected for biomarkers and histological analysis. For BALF collection, lungs were gently washed three times using a cannula inserted into the trachea with 0.6 mL of solution [1X HBSS (Hanks’ Balanced Salt Solution), 10 mM EDTA (Ethylenediaminetetraacetic acid; Fluka analytical, Sigma-Aldrich, St. Louis, MO, United States), 10 mM HEPES ((4-(2-hydroxyethyl)-1-piperazineethanesulfonic acid; Gibco, ThermoFisher Scientific, Waltham, MA, United States) and distilled water]. Routine recovery of BALF did not significantly differ among animals with ∼80% of instilled volume recovered. The obtained BALF was centrifuged at 1,000 g and 4°C for 10 min and the cell-free supernatant was aliquoted and stored at −80°C for quantitative determination of the biomarkers.

After BALF procedure and cardiac perfusion, the whole lungs were removed, inflated with a cannula through the trachea by gentle infusion with 0.6 mL of 10% formalin solution (Formaldehyde solution 4%, buffered, pH 6.9; Sigma-Aldrich, St. Louis, MO, United States) and fixed for at least 24 h at room temperature. After fixation, lungs were dehydrated in graded ethanol series, clarified in xylene and paraffin embedded. Each longitudinally oriented lung was cut to obtain one representative 5 µm thick slice, which subsequently were stained with Masson’s Trichrome histological staining.

### Biomarker analysis

The total amount of MMP-7 was quantified in BALF samples with commercial Enzyme-linked immune-sorbent assay (ELISA) kits specific for mouse proteins according to the manufacturer’s protocol (NovusBiologicals, NBP3-06895). Results were expressed as pg of protein per mL of sample.

### Histological analysis

**FIGURE 2:**
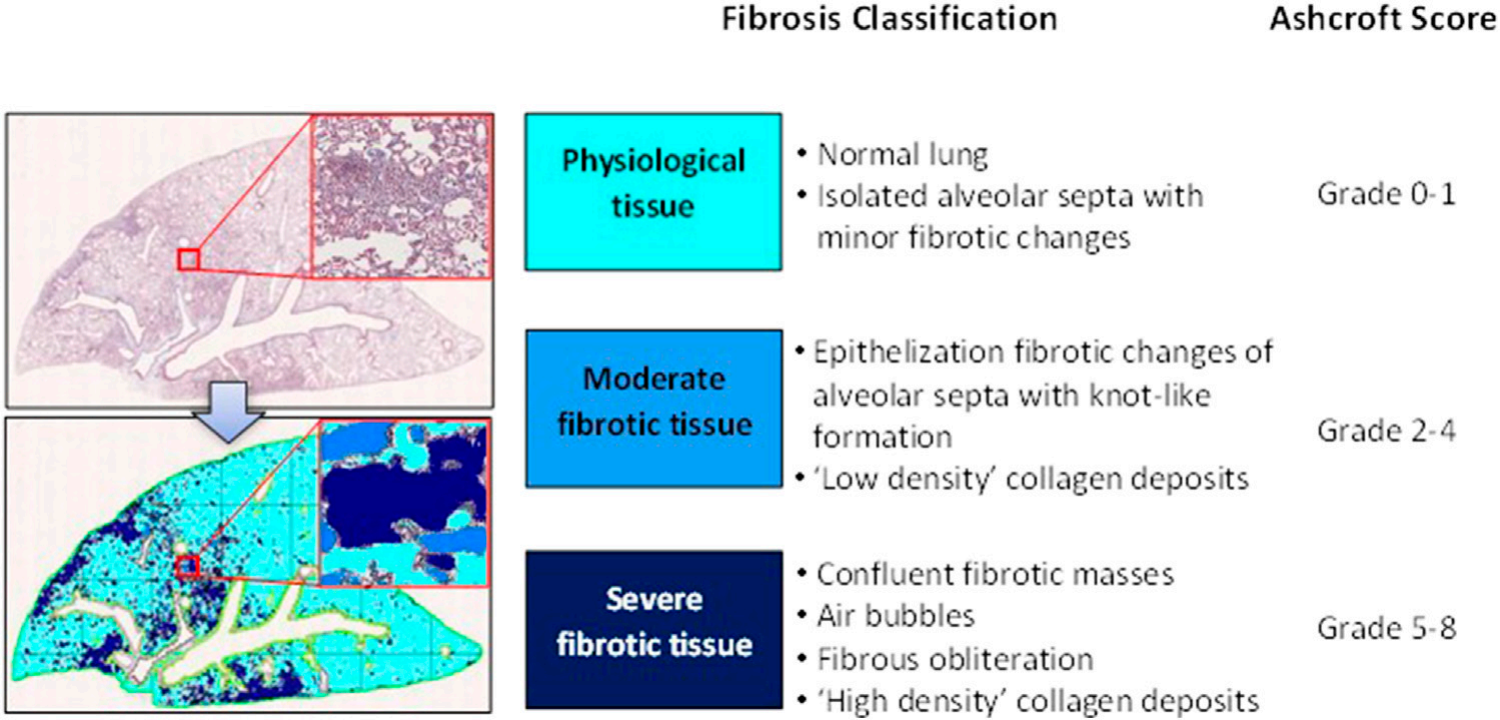
Schematic classification of lung fibrosis severity distribution by AI-based Visiopharm^®^ APPs.

For histological analysis, Masson’s trichrome stained slides representative of all the five lobes were digitalized through the image analysis system NDP. scan 3.2 with NanoZoomer S-60 scanner (Hamamatsu Photonics K.K, Japan). Scans were performed using the fully automated mode and ×20 objective. Fibrotic lung injury was blindly assessed on the whole-slide images through Ashcroft scoring system from grade 0 to 8 (Hübner et al., 2008). Ashcroft score for each animal was expressed as the mean of the individual scores observed throughout all the analyzed fields. The more conventional histopathological analysis assessed by Ashcroft scoring was associated with an automated image analysis that was able to assess different grades of fibrosis severity on whole-slide images with the support of the AI deep learning Visiopharm^®^ software. The AI-based APP was developed for the purpose of recognizing and classifying different degrees of fibrosis severity corresponding to the different grades described in the modified Ashcroft scale (Pontis et al., 2019). Specifically, in addition to identifying the lung parenchyma by excluding the basal collagen, main bronchi and blood cells, as described previously, this APP was designed to distinguish between physiological tissue and fibrotic regions and classify them as moderate and severe fibrosis (Figure 2).

### Statistical analysis

All data were expressed as mean ± standard error of mean (SEM) of 12–25 observations. Unpaired *t*-test or analysis of variance (ANOVA) followed by Sidak’s or Dunnett’s *post hoc* analysis was used to compare two or more experimental groups, respectively. The Pearson’s correlation coefficient matrix (r value) was used to determine the degree of association between data.

Statistical analyses were performed using GraphPad Prism 9 software (GraphPad Software Inc., San Diego, CA, United States). *p*-value ≤0.05 was considered statistically significant.

## Results

To validate FVC and DFCO as novel functional readouts representative of the pathological progression of pulmonary fibrosis in the BLM model, the effect of Nintedanib and Pirfenidone therapeutic treatments were tested in two separate studies following the protocol described in the “Materials and Methods” sections.

Nintedanib and Pirfenidone were administered orally respectively at the dose of 50 mg/kg b.i.d. and 200 mg/kg b.i.d. according to the protocol previously described.

**FIGURE 3:**
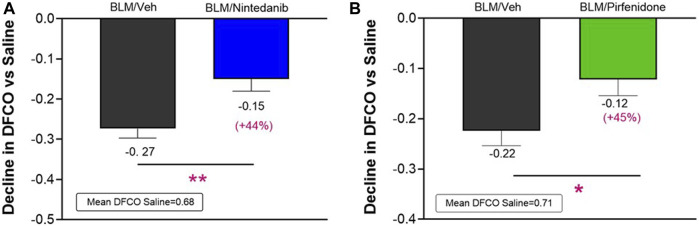
Effect of Nintedanib **(A)** and Pirfenidone **(B)** on DFCO decline at day 21 after the first BLM OA administration in mice. Data represent mean ± SEM values for BLM/Veh (*n* = 25 for Nintedanib study and *n* = 23 for Pirfenidone study; black bar) and BLM/Nintedanib (*n* = 21; blue bar) or BLM/Pirfenidone (*n* = 24; green bar) groups of animals expressed as difference vs*.* mean of Saline group (*n* = 12 for each study). Statistical analysis was assessed using unpaired Student’s *t*-test vs*.* the BLM group; *p ≤ 0.05, **p ≤ 0.01. BLM, bleomycin; Veh, vehicle.

At day 21 BLM treatment alone led to a significant reduction in DFCO of −0.27 ± 0.02 and of −0.22 ± 0.03 compared to control animals (mean saline DFCO: 0.68 ± 0.02 and 0.71 ± 0.02) in Nintedanib and Pirfenidone studies, respectively. Nintedanib and Pirfenidone treatments significantly slowed down the decline in DFCO value by 44% (Figure 3A) and 45% (Figure 3B), respectively (Nintedanib group decline of 0.15 ± 0.03; difference vs*.* BLM: 0.12, *p* ≤ 0.01 and Pirfenidone group decline of 0.12 ± 0.03; difference vs*.* BLM: 0.10, *p* ≤ 0.05).

**FIGURE 4:**
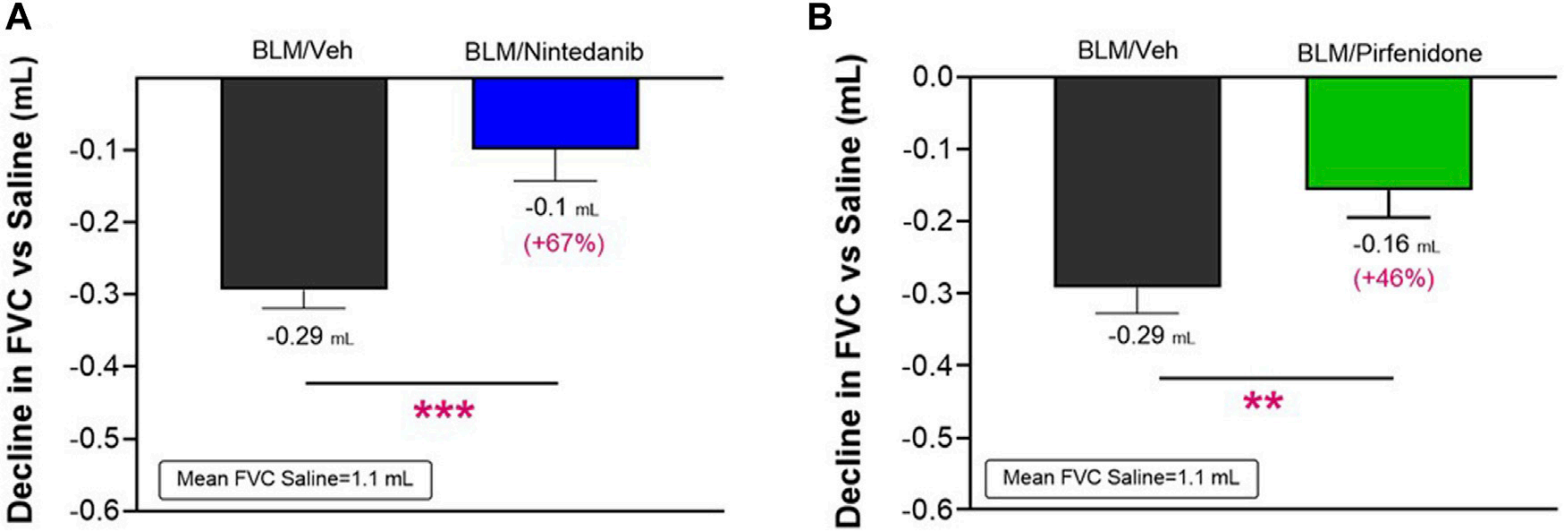
Effect of Nintedanib **(A)** and Pirfenidone **(B)** on the decline in FVC at day 21 after the first BLM OA administration in mice. Data represent mean ± SEM values for BLM/Veh (*n* = 25 for Nintedanib study and *n* = 23 for Pirfenidone study; black bar) and BLM/Nintedanib (n = 21; blue bar) or BLM/Pirfenidone (*n* = 24; green bar) groups of animals. Data are expressed as difference of mL vs*.* the observed mean of the Saline group (*n* = 12 for each study). Statistical analysis was carried out using unpaired Student’s *t*-test in comparison with the BLM group; **p ≤ 0.01, ***p ≤ 0.001. BLM, bleomycin; Veh, vehicle.

The lung function tests were performed on day 21 after the first BLM OA administration by FlexiVent System. As observed in clinical practice, FVC resulted in the most affected parameter among those calculated by instrument for this disease model (data not shown). Consistent with an expected increase in lung stiffness and a restrictive airway pattern typical of the fibrotic disease, a decline in FVC of 0.29 ± 0.02 mL (Nintedanib study) and of 0.29 ± 0.03 mL (Pirfenidone study) compared to the respective Saline groups (mean FVC: 1.1 ± 0.02 mL and 1.1 ± 0.03 mL) was observed in BLM mice in both studies. At day 21, Nintedanib treatment significantly reduced BLM-induced alterations in lung function, significantly improving the FVC by 67% when compared with the BLM group of mice (observed decline of 0.1 ± 0.04 mL; difference vs*.* BLM: 0.19 mL, *p* ≤ 0.001) (Figure 4A). Similarly, Pirfenidone was able to significantly reduce the FVC decline by 46% when compared to BLM group (0.16 ± 0.04 mL; difference vs*.* BLM: 0.13 mL, *p* ≤ 0.01) (Figure 4B).

**FIGURE 5:**
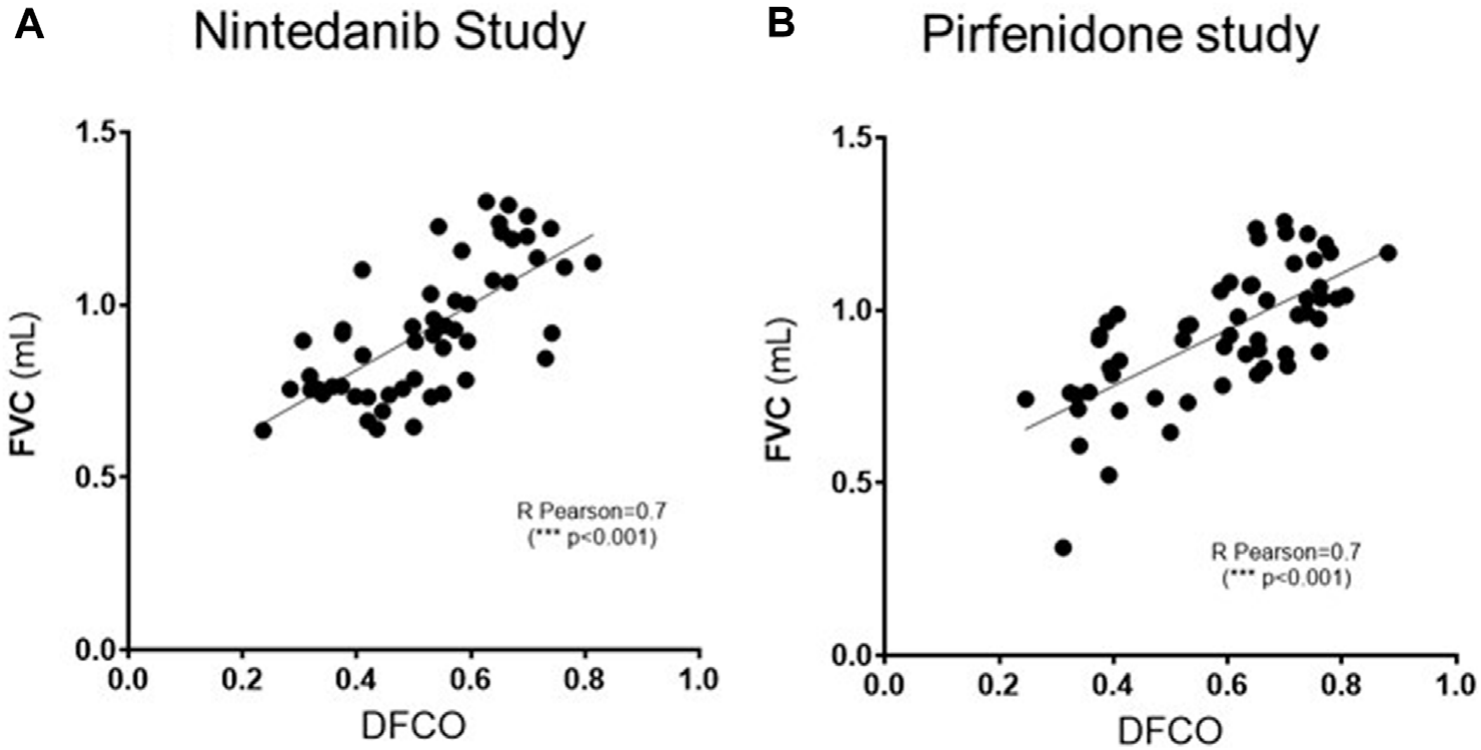
Pearson’s correlation coefficient and linear regression analysis of the relationship between FVC (mL) and DFCO among all the experimental groups (Saline, Bleomycin, Nintedanib or Pirfenidone) in Nintedanib **(A)** and Pirfenidone **(B)** studies. ***p ≤ 0.001.

As shown in Figure 5, a significant correlation (R Pearson = 0.7, ***p ≤ 0.001 for both studies) was observed between FVC and DFCO values in all the animals of the different experimental groups.

**FIGURE 6:**
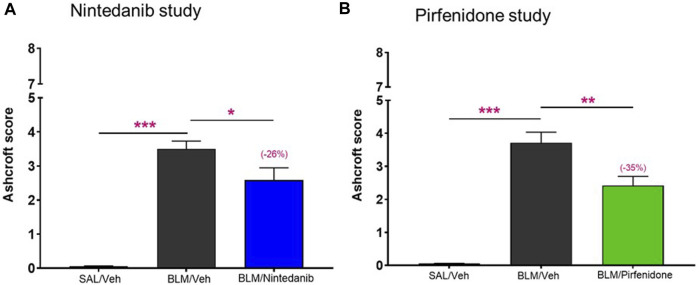
Effect of Nintedanib **(A)** and Pirfenidone **(B)** on Ashcroft score. Data represent mean ± SEM values for SAL/Veh (*n* = 12 for each study; white bar), BLM/Veh (*n* = 25 for Nintedanib study and *n* = 23 for Pirfenidone study; black bar) and BLM/Nintedanib (*n* = 21; blue bar) or BLM/Pirfenidone (*n* = 24; green bar) groups of animals. Statistical analysis was assessed using one-way ANOVA followed by Dunnett’s test in comparison with the BLM group; ***p ≤ 0.001, **p ≤ 0.01, *p ≤ 0.05. SAL, Saline; BLM, bleomycin; Veh, vehicle.

To further validate these novel readouts, we compared them with the results obtained from the histological analysis commonly and routinely carried out in preclinical models of pulmonary fibrosis. The Ashcroft score, widely used method to quantify pulmonary fibrosis based on histological analysis, showed a significant increase in the BLM mice when compared to control mice treated with Saline in both studies (3.5 ± 0.23 vs*.* 0.06 ± 0.01, *p* ≤ 0.001 for the Nintedanib study and 3.71 ± 0.32 vs*.* 0.05 ± 0.01, *p* ≤ 0.001 for the Pirfenidone study). Nintedanib 50 mg/kg b. i.d. significantly reduced Ashcroft scores when compared with the BLM group (2.58 ± 0.36, 26% of inhibition vs*.* BLM group, *p* ≤ 0.05) (Figure 6A). Similarly, the treatment with Pirfenidone at the dose of 200 mg/kg b.i.d. significantly reduced the value of Ashcroft score when compared with the BLM group (2.42 ± 0.28, 35% of inhibition vs*.* BLM group, *p* ≤ 0.01) (Figure 6B).

**FIGURE 7:**
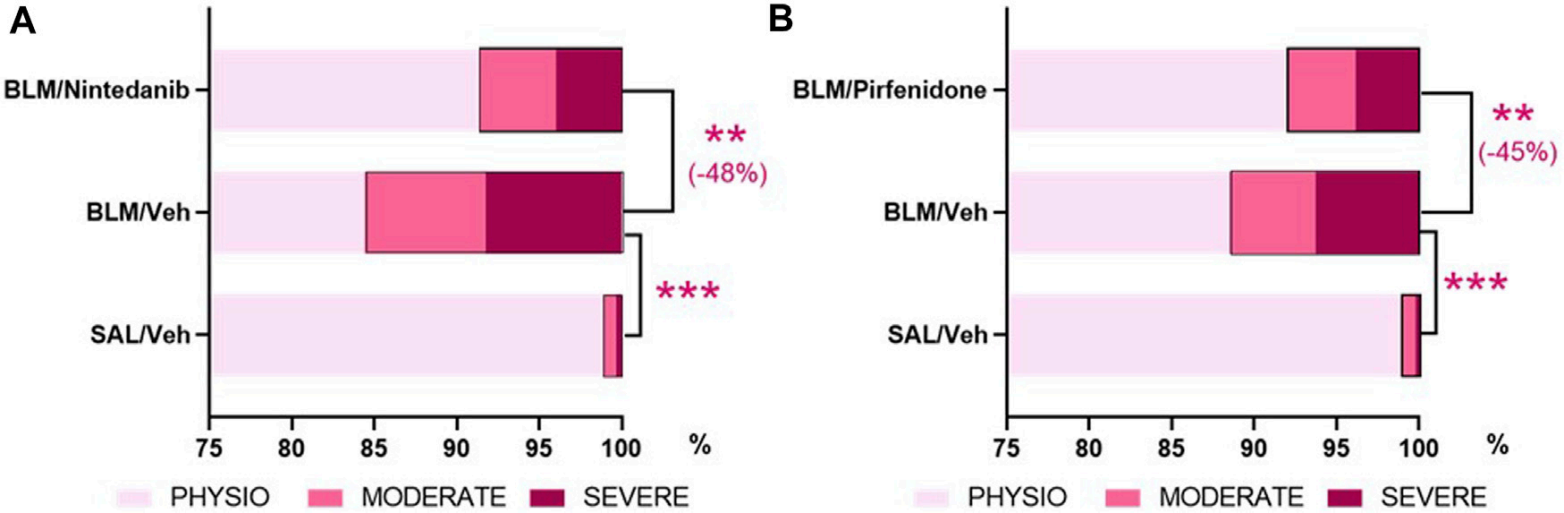
Effect of Nintedanib **(A)** and Pirfenidone **(B)** on tissue classification of lung fibrosis severity distribution using AI-based Visiopharm^®^ APPs. Data represent mean ± SEM values for BLM/Veh (*n* = 25 for Nintedanib study and *n* = 23 for Pirfenidone study) and BLM/Nintedanib (*n* = 21) or BLM/Pirfenidone (*n* = 24) groups of animals. Statistical analysis was assessed using one-way ANOVA followed by Dunnett’s test in comparison with the BLM group; ***p ≤ 0.001, **p ≤ 0.01. BLM, bleomycin; Veh, vehicle.

The AI-based Visiopharm^®^ APPs analysis, developed in order to reduce the intrinsic limitations of the Ashcroft score as an operator-assessed measurement, provided additional confirmation that both Nintedanib (Figure 7A) and Pirfenidone (Figure 7B) were able to significantly reduce the percentage of severe and moderate fibrotic tissues with a parallel increase of physiological tissue when compared to the BLM group (severe fibrosis: Nintedanib 4.0± 0.7% vs*.* BLM 8.3± 1.2%, moderate fibrosis: Nintedanib 4.7± 0.8% vs*.* BLM 7.3± 0.7%, physiological tissue: Nintedanib 91.4± 1.4% vs*.* BLM 84.4 ± 1.8%; severe fibrosis: Pirfenidone 3.9 ± 0.8% vs*.* BLM 8.6 ± 1.4%, moderate fibrosis: Pirfenidone 4.1 ± 0.5% vs*.* BLM 5.1 ± 0.7%, physiological tissue: Pirfenidone 92.0 ± 1.3% vs*.* BLM 88.8 ± 1.9%). The control group treated with Saline, as expected, mostly showed physiological tissue, with statistically significant differences vs*.* the BLM group in all three different tissue classifications (physiological, moderate, and severe). Consolidating the values of the fibrotic lesions (moderate plus severe tissue), Nintedanib was able to reduce the lesions by 48% when compared to BLM group (moderate and severe lesions Nintedanib: 8.6 ± 1.4% vs*.* BLM 15.6 ± 1.8%; *p* < 0.01). Similarly, Pirfenidone reduced the lesions by 45% when compared to BLM group (moderate plus severe tissue Pirfendone: 8.0 ± 1.3% vs*.* BLM 13.7 ± 2.2%; *p* < 0.01). The percentage of fibrotic tissue in the saline groups of the two studies was very low (1.1 ± 0.2% in the Nintedanib study and 1.0 ± 0.2% in the Pirfenidone study) and, as expected, statistically significant differences were observed when compared to BLM mice (*p* < 0.001 for the Nintedanib study and *p* < 0.001 for the Pirfenidone study).

**FIGURE 8:**
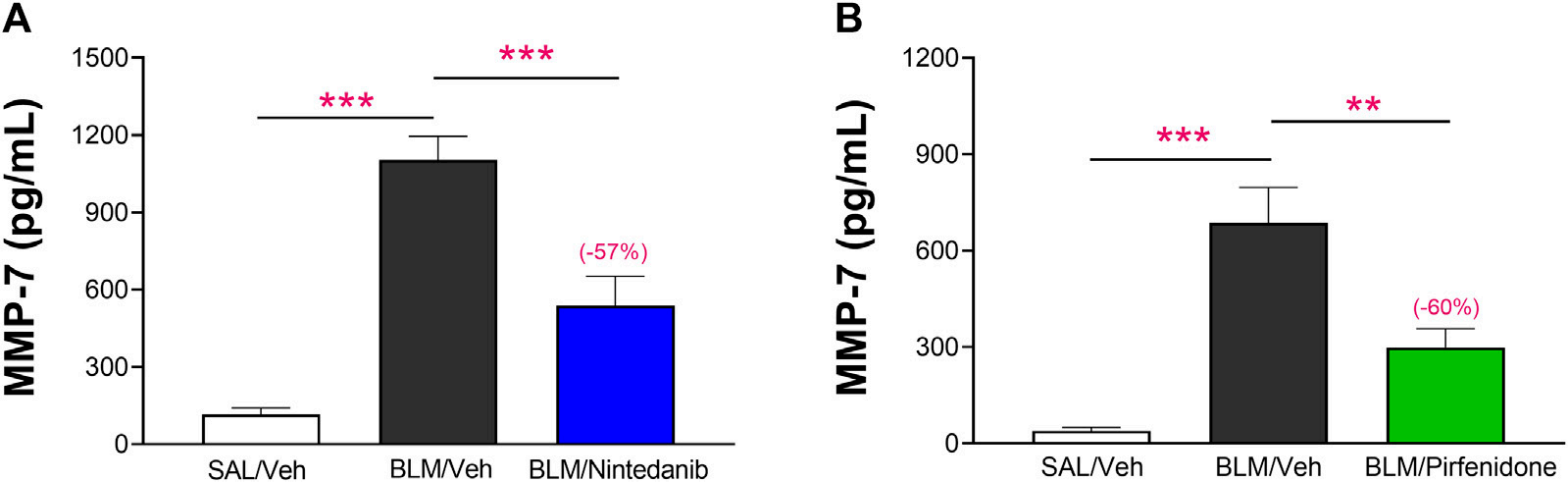
Effect of Nintedanib **(A)** and Pirfenidone **(B)** on the levels of MMP-7 in BALF. Data represent mean ± SEM values for SAL/Veh (*n* = 12 for each study; white bar), BLM/Veh (*n* = 25 for Nintedanib study and *n* = 23 for Pirfenidone study; black bar) and BLM/Nintedanib (*n* = 21; blue bar) or BLM/Pirfenidone (*n* = 24; green bar) groups of animals. Statistical analysis was assessed using one-way ANOVA followed by Dunnett’s test in comparison with the BLM group; ***p ≤ 0.001, **p ≤ 0.01, *p ≤ 0.05. BLM, bleomycin; Veh, vehicle.

The added value and robustness of the novel functional readouts DLCO and FVC were finally assessed comparing the results obtained with the BALF levels of MMP-7, a prognostic biomarker of lung fibrosis in clinical studies (Maher et al., 2023). The results obtained showed that the concentrations of MMP-7 at day 21 were significantly elevated in BALF of BLM-treated animals when compared to Saline-control animals (1,103 ± 92 pg/mL vs*.* 116 ± 25 pg/mL, *p* ≤ 0.001 in the Nintedanib study and 686 ± 110 pg/mL vs*.* 39 ± 11 pg/mL, *p* ≤ 0.001 in the Pirfenidone study) and that Nintedanib and Pirfenidone treatments significantly reduced MMP-7 in BALF (538 ± 114 pg/mL, −57% vs*.* BLM group; *p* ≤ 0.001 and 298 ± 59 pg/mL, −60% vs*.* BLM group; *p* ≤ 0.01, respectively) (Figures 8A, B).

**FIGURE 9:**
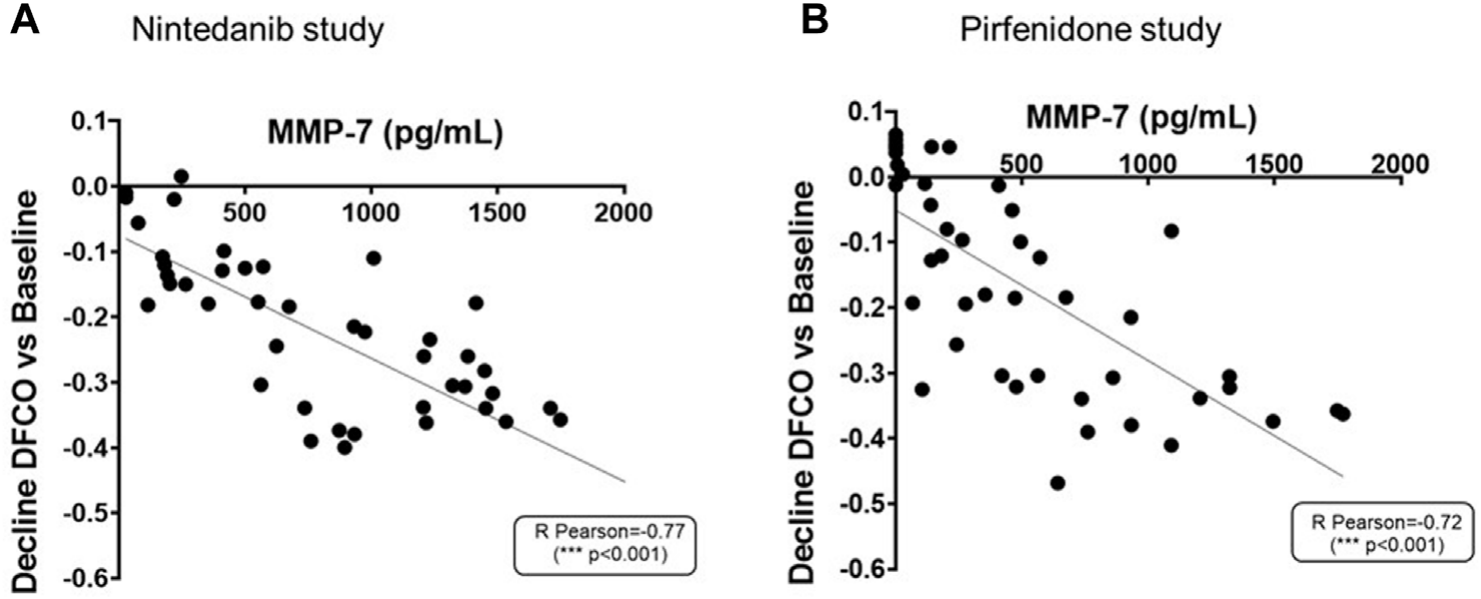
Correlation between MMP-7 levels in BALF and the decline of DFCO assessed in both Nintedanib **(A)** and Pirfenidone **(B)** studies. The correlation between MMP-7 (pg/mL) and decline of DLCO was carried out by Pearson’s correlation coefficient and linear regression analysis ***p ≤ 0.001.

As shown in Figures 9A, B, increased concentrations of MMP-7 in BALF were associated with more pronounced declines of DFCO (R Pearson = −0.77 and −0.72; *p* < 0.001 for Nintedanib and Pirfenidone studies, respectively).

As a final and summary step, the correlations among indicators of lung function, histology, DFCO, and the fibrotic biomarker (MMP-7) were also analyzed for both studies.

**FIGURE 10:**
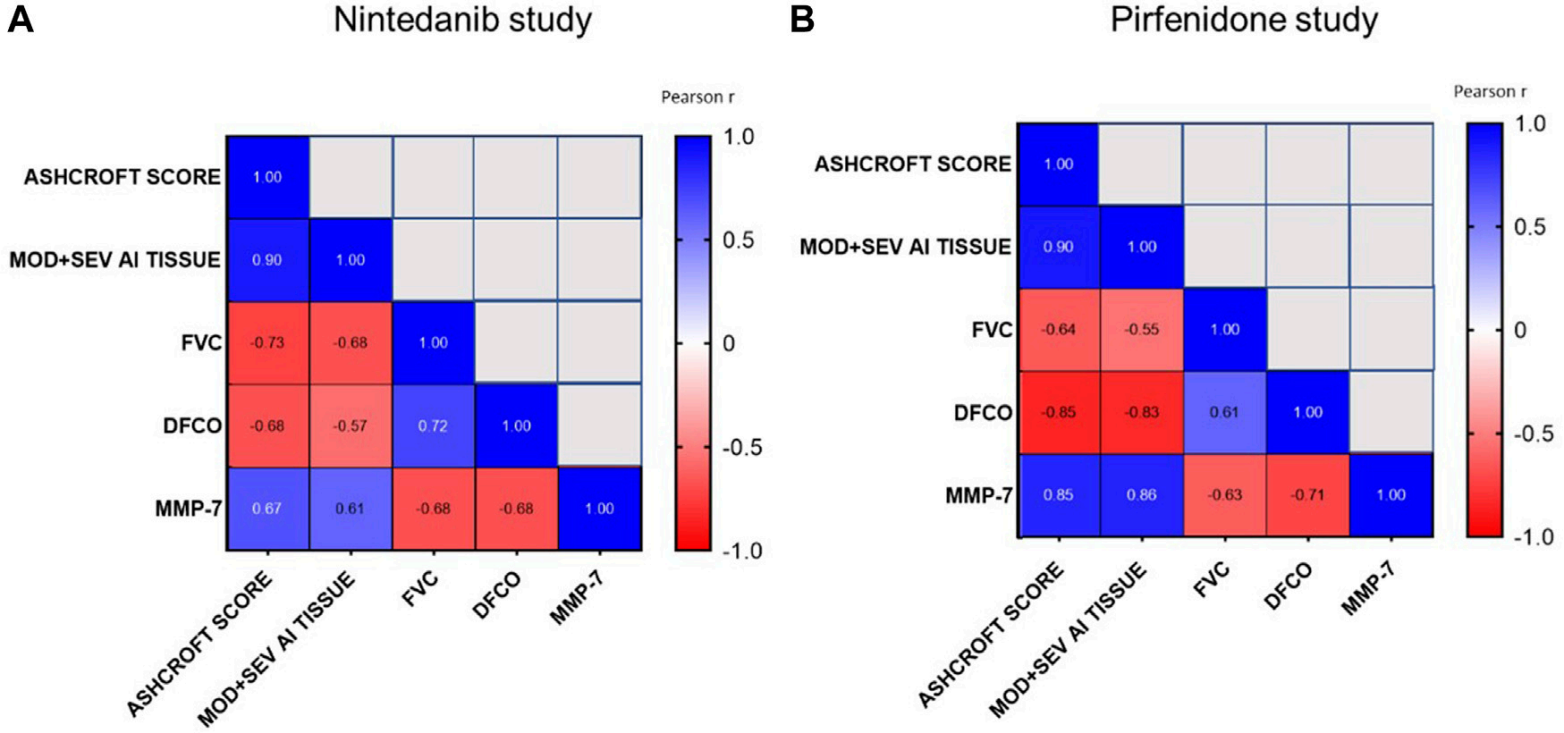
Correlations between Ashcroft score analysis, moderate and severe fibrotic tissue by AI, FVC, DFCO and MMP-7 at day 21 after BLM OA administration in mice treated with Nintedanib **(A)** or Pirfenidone **(B)** or vehicle. Correlations were determined using Pearson’s correlation coefficient matrix tests.

As shown in Figures 10A, B, the development of lung fibrosis was accompanied by obstruction of the airways and reduced alveolar gas exchange as indicated by the negative correlation of functional readouts such as FVC and DFCO with Ashcroft score (Pearson R = −0.73 and R = −0.68 respectively for FVC and DFCO in the Nintedanib study and Pearson R = −0.64 and R = −0.85 respectively for FVC and DFCO in the Pirfenidone study) and the moderate and severe fibrotic lesions detectable by AI (Pearson R = −0.68 and R = −0.57 respectively for FVC and DFCO in Nintedanib study and Pearson R = −0.55 and R = −0.83 respectively for FVC and DFCO in the Pirfenidone study, *p* < 0.001). On the other hand, strong positive correlations were observed between changes of the two functional parameters (Pearson R = 0.72 and R = 0.61 between FVC and DFCO for Nintedanib and Pirfenidone studies, respectively) as well as between the two separate evaluations of histologically assessed lung fibrosis (Pearson R = 0.90 and Pearson R = 0.90 between Ashcroft scores and the severe and moderate lesions evaluated by AI in Nintedanib and Pirfenidone studies, respectively).

Finally, concentrations of MMP-7 in BALF, positively correlated with Ashcroft score and AI (Pearson R = 0.67 and R = 0.61 respectively, in the Nintedanib study and R = 0.85 and R = 0.86 respectively, *p* < 0.001 in the Pirfenidone study) and negatively correlated with FVC and DFCO (Pearson R = −0.68 and R = −0.68 respectively, in the Nintedanib study and Pearson R = −0.63 and R = −0.71 respectively, *p* < 0.001 in the Pirfenidone study), providing additional support to the relevance of the novel readouts assessed in this study.

## Discussion

IPF is a fatal and complex disease, with limited therapeutic options for which novel approaches need to speed up and a more effective drug development process is necessary (White et al., 2022). While many anti-fibrotic compounds showed promising results in preclinical models of pulmonary fibrosis (Jenkins et al., 2017), the development of these drugs is beset by a high attrition rate. This has led to discuss actively about the translational power of preclinical models that despite showing some intrinsic limits, still represent a critical step for the selection of drug candidates for clinical development. In order to develop a robust preclinical model with improved reliability for preclinical screening of novel therapeutic approaches, the introduction of clinically meaningful readouts could be very relevant. Lung function assessment is recognized as an important tool to evaluate the mouse respiratory disease models, but it is rarely used because the equipment necessary is expensive and requires very specific technical competency.

Despite some limitations such as the progressive resolution of the fibrosis over the time and that does not recapitulate all the features of human disease, the BLM model remains the most common and important animal model of lung fibrosis, and it is considered a good tool to assess efficacy of new anti-fibrotic compounds. To improve the translational potential of the BLM model (Stellari et al., 2017), we have therefore explored the use of lung function tests such as FVC and the assessment of gas exchange at alveolar level using the DFCO**.** The assessment of lung function through the determination of FVC represents the first step in the diagnosis and in the monitoring of IPF progression (Raghu et al., 2022). An absolute or relative decline in the % predicted FVC ≥10% is associated with mortality (Durheim et al., 2015; Paterniti et al., 2017), changes in FVC decline were selected as primary endpoints in the pivotal phase 3 trials of antifibrotic therapies (Noble et al., 2011; King et al., 2014; Richeldi et al., 2014). In experimental animals, pulmonary function is generally assessed using the forced oscillation technique (FOT). This is an invasive method allowing accurate measurements of physiologically relevant parameters describing the mechanical properties of the respiratory system. A classic approach to the assessment of lung mechanics in animals is the measurement of dynamic lung resistance (RL) and compliance (Cdyn). Changes in these respiratory mechanics parameters reflect changes in the resistance to the air moving in and out of the lungs and in the distensibility of the respiratory system, respectively, but it is not always clear how they associate to the commonly measured forced expiration (FE) parameters typically assessed in humans (Ranu et al., 2011). Nowadays, spirometric-like measures can be performed in mice by rapidly exposing the airways to a negative pressure to generate a forced expiratory flow signal (Shalaby et al., 2010). The technique is referred to as negative pressure-driven forced expiration (NPFE) and it can be performed concurrently with FOT measurements, in the same animal using a single instrument, i.e., the FlexiVent system (Scireq, EMKA Technologies). This approach allows to obtain, in addition to respiratory mechanics’ parameters, endpoints resembling the clinically used ones, such as FVC. In our model, 21 days after the first BLM administration, a decline in FVC was indeed observed when compared to values observed in the saline group, and this decline was markedly reduced by therapeutic treatment with both Nintedanib and Pirfenidone.

DFCO measurement directly reflects pulmonary structural changes (Pastre et al., 2021) and it was found reduced by 40% on average at the time of initial evaluation in IPF patients (Cortes-Telles et al., 2014), while strongly correlating with dyspnea (Swigris et al., 2012) and survival (Hamada et al., 2007). Despite its ubiquitous use in clinical trials and practice, DFCO has rarely been evaluated in murine models of lung diseases. Following the procedure described by Fallica and coll. (Fallica et al., 2011). we measured DFCO in our model at different timepoints after the BLM injury, and we were able to pair the effective decline of this measurement with development and progression of pulmonary fibrotic lesions; we showed that DFCO changes over time can be pharmacologically modulated by treating the animals with the IPF drugs Nintedanib and Pirfenidone, that administered orally, significantly increased DFCO values at 21 days after BLM, confirming the ability of these drugs to improve alveolar gas exchange.

Despite the clinical relevance of DFCO and FVC, at preclinical level the histological analysis is still considered the gold standard and widely used in most preclinical studies. We therefore compared the results obtained with DFCO and FVC with the results of the histological analysis, performed both by the widely used Ashcroft score (Hübner et al., 2008) and by a novel, automated image analysis developed with the support of AI (Pontis et al., 2019). As expected, Nintedanib and Pirfenidone were able to reduce the Ashcroft score in a statistically significant manner, a result that was even more apparent when assessed by automated image analysis, that was able to separately quantitate moderate and severe fibrosis. The strong correlation between functional and histological readouts, with DFCO and FVC improvements mirroring the decrease observed in Ashcroft scores and in moderate and severe fibrotic tissues detected by AI, further validated the robustness of the functional readouts introduced in our studies.

Finally, it is also important to underscore the correlations observed between changes in functional parameters and MMP-7, a novel biomarker of ECM remodeling, included in the PROFILE (Prospective Study of Fibrosis in Lung Endpoints) study (Maher et al., 2017) as a measurement that may allow to follow the progression and the prognosis in IPF patients (Saini et al., 2012). MMP-7 levels were found to be elevated in plasma, BALF, and lung tissue of IPF patients as compared to healthy controls, and their differential concentrations appeared to distinguish IPF patients from those with chronic respiratory disorders (Rosas et al., 2008) or other ILDs (Morais et al., 2015). Indeed, MMP-7 is now considered one of the most promising prognostic biomarkers in IPF, with circulating levels correlating with disease severity, as assessed by FVC and DFCO, and with survival, as reported in several studies (Inoue et al., 2020). The inverse correlations observed between both FVC and DFCO and the levels of MMP-7 in BALF, either in the absence or presence of antifibrotic treatments, provide a final support to the capability of FVC and DFCO to properly represent disease progression in the BLM mouse model of IPF.

Taking together all these results, we demonstrated for the first time the intrinsic value of evaluating clinically relevant readouts in a preclinical model of pulmonary fibrosis, showing their potential ability to better catch the efficacy of new anti-fibrotic treatments, making the BLM-induced fibrosis in mice a robust and more predictive model for drugs screening in IPF.

Drug discovery in IPF is proving very challenging as a consequence of IPF being a very complex disease, difficult to fully understand and to mimic with animal models. Considering these intrinsic limitations, the goal of this paper was to generate a more reliable, predictive and robust animal model of pulmonary fibrosis by introducing clinically relevant endpoints, not commonly evaluated in preclinical studies, with the aim of bringing the model closer to human disease and more suitable for the identification of new anti-fibrotic drugs that would result active in man.

The assessment of pulmonary functions, not commonly performed in preclinical studies aimed at the determination of the efficacy of novel anti-fibrotic compounds, should therefore be considered as an important tool with a high translational value, given its relevance in clinical trials and in the management of IPF patients. The results of the present study demonstrate not only that in mice it is possible to evaluate the same functional parameters assessed in patients with IPF, but also that they can provide an accurate profile of a compound’s anti-fibrotic activity, as shown by the results obtained with Nintedanib and Pirfenidone; both drugs were in fact capable to modulate significantly the changes in FVC and DFCO induced by BLM, in keeping with the clinical data. Moreover, these readouts are reproducible across studies, more sensitive when compared to histological analysis, and require less time-consuming processing.

We believe that the present work casts a new light on the importance to examine clinically relevant endpoints in preclinical studies and may provide a novel drug discovery approach in IPF, with the final goal to improve the selection process of new anti-fibrotic candidates for clinical studies and reduce the currently high attrition rate of novel treatments for IPF.

## Data Availability

The original contributions presented in the study are included in the article/supplementary material, further inquiries can be directed to the corresponding author.
